# MicroRNA-29a induces loss of 5-hydroxymethylcytosine and promotes metastasis of hepatocellular carcinoma through a TET–SOCS1–MMP9 signaling axis

**DOI:** 10.1038/cddis.2017.142

**Published:** 2017-06-29

**Authors:** Qing Chen, Dan Yin, Yong Zhang, Lei Yu, Xue-Dong Li, Zheng-Jun Zhou, Shao-Lai Zhou, Dong-Mei Gao, Jie Hu, Cheng Jin, Zheng Wang, Ying-Hong Shi, Ya Cao, Jia Fan, Zhi Dai, Jian Zhou

**Affiliations:** 1 Key Laboratory of Carcinogenesis and Cancer Invasion, Liver Cancer Institute, Zhongshan Hospital, Fudan University, Ministry of Education, Shanghai 200032, China; 2 Institute of Biomedical Sciences, Fudan University, Shanghai 200032, China; 3 Key Laboratory of Carcinogenesis and Cancer Invasion, Cancer Research Institute, Central South University, Ministry of Education, Changsha 410078, China; 4 State Key Laboratory of Genetic Engineering, Fudan University, Shanghai 200032, China

## Abstract

Ten eleven translocation (TET) enzymes convert 5-methylcytosine (5-mC) to 5-hydroxy-methylcytosine (5-hmC) and have crucial roles in biological and pathological processes by mediating DNA demethylation, however, the functional role of this epigenetic mark and the related enzymes in hepatocellular carcinoma (HCC) progression remains unknown. Here, we demonstrated that TET-family enzymes downregulation was one likely mechanism underlying 5-hmC loss in HCC. We found that miR-29a overexpression increased DNA methylation of suppressor of cytokine signaling 1 (SOCS1) promoter was associated with HCC metastasis *in vitro* and *in vivo*. Furthermore, miR-29a silenced anti-metastatic SOCS1 through direct TET-family targeting, resulting in SOCS1 promoter demethylation inhibition. Chromatin immunoprecipitation analyses confirmed that TET1 regulated SOCS1 expression through binding to the promoter region of SOCS1. Finally, miR-29a overexpression correlated with poor clinical outcomes and TET–SOCS1–matrix metalloproteinase (MMP) 9 axis silencing in HCC patients. In conclusion, our findings demonstrate that 5-hmC loss is an epigenetic hallmark of HCC, and miR-29a is an important epigenetic modifier, promoting HCC metastasis through TET–SOCS1–MMP9 axis silencing. The results offer a new strategy for epigenetic cancer therapy.

Hepatocellular carcinoma (HCC) is one of the most common cancers worldwide and is among the leading causes of cancer-related death, especially in China.^1^ As HCC is often diagnosed at an advanced stage, a large proportion of HCC patients display symptoms of intrahepatic metastases or experience postsurgical recurrence, and have a 5-year survival rate of ~30–40%.^2^ Therefore, identifying novel HCC progression molecular markers and gaining insight into the molecular mechanisms responsible for metastasis and postsurgical recurrence will contribute to cancer prevention and increased life expectancy for HCC patients.

Abnormal DNA methylation at 5 position of cytosine (5-mC) is a well-known cancer epigenetic feature. Recent studies demonstrated that promoter hypermethylation and subsequent inactivation of tumor suppressor genes were involved in liver carcinogenesis.^3, 4^ Therefore, specific DNA methylation patterns alterations are hallmarks of tumors and could represent specific HCC treatment targets.^5^ The recent discovery of ten eleven translocation (TET) family members that specifically modify DNA via 5-mC hydroxylation may explain how cells erase existing methylation marks.^6, 7^ Within the TET proteins family, TET1, 2, and 3 have been shown to convert 5-mC to 5-hydroxy-methylcytosine (5-hmC), which exists at a high level in self-renewing, pluripotent stem cells.^6, 8^ While, 5-hmC levels are profoundly reduced in many tumors types including breast cancer, liver cancer, and colon cancer.^9, 10^ These findings identified 5-hmC as a tumor development-associated biomarker. Furthermore, TET2 mutational inactivation and deletion have been associated with decreased 5-hmC levels in various hematopoietic malignancies.^11, 12^ Taken together, these studies suggest a crucial role for TET enzymes and 5-hmC in malignancy.

MicroRNAs (miRNAs) comprise an abundant class of small, highly conserved noncoding RNAs that bind the 3′-UTRs of protein-coding genes to suppress gene expression.^13^ miRNAs can act as oncogenic promoters or tumor suppressors, depending on their target gene’s function within a specific cell or tissue type.^14, 15^ Song *et al.*^16^ demonstrated that miR-22 exerts its metastatic potential by silencing anti-metastatic miR-200 through direct targeting of the TET protein family, thereby inhibiting miR-200 promoter demethylation. This finding highlights the link between miRNA, TET proteins, and DNA demethylation during tumor progression; however, the role of miRNAs in TET expression and active DNA demethylation in liver carcinogenesis and metastasis remains unclear.

Here, we report that 5-hmC loss serves as an epigenetic hallmark for HCC. Our findings indicate that 5-hmC, TET1, and TET3 significantly impact HCC progression. Importantly, we demonstrate that miR-29a promotes metastasis through SOCS1-expression repression and 5-hmC modulation by directly targeting TET-family members. Ultimately, these findings contribute to our current understanding of cancer epigenetics.

## Results

### Loss of 5-hmC correlates with HCC metastasis and prognosis

**Figure 1:**
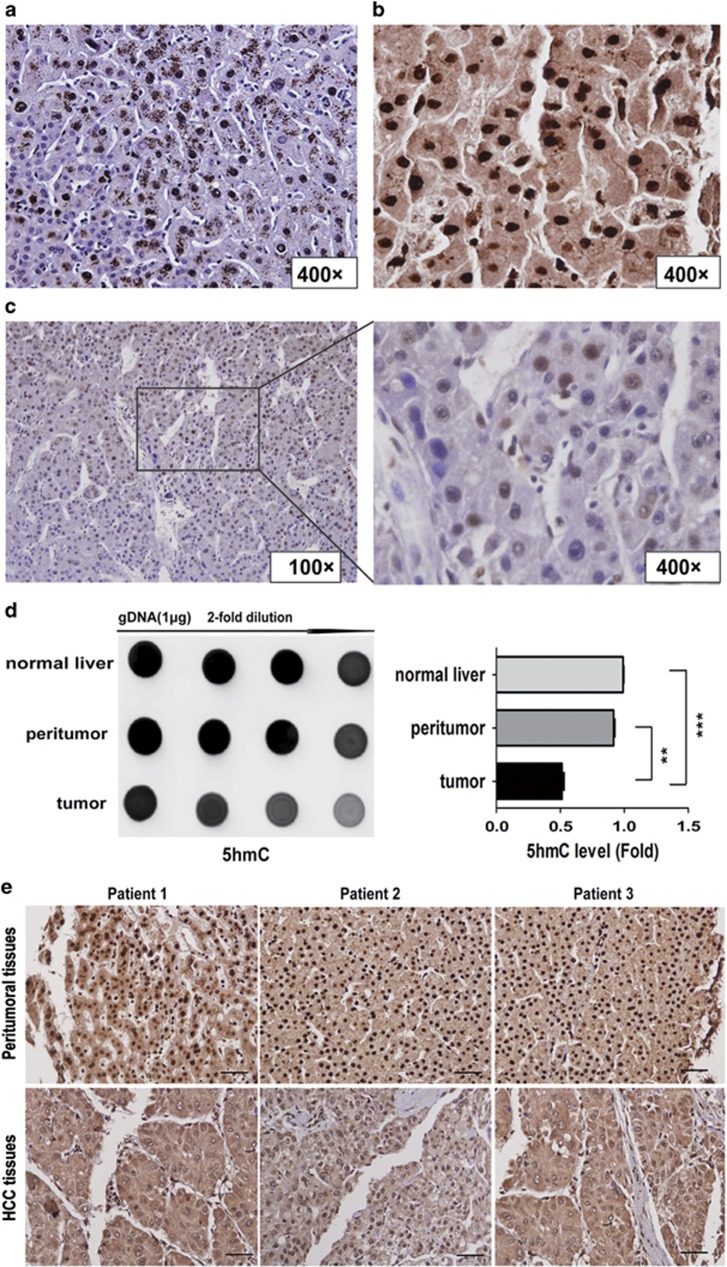

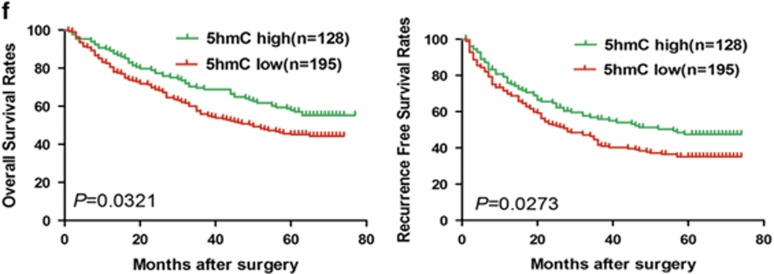
5-hmC loss correlates with HCC progression. (**a–c**) Representative histology of 5-hmC IHC staining in normal liver (**a**), peritumor (**b**), and HCC tissues(**c**). (**d**) Global 5-hmC levels among normal, peritumoral and tumoral tissues, the 5-hmC levels were quantified. Data are shown as mean±S.D. (*n*=3). **P<0.01, ***P<0.001. (**e**) Representative immuostaining images of 5-hmC in HCC TMA (*n*=323). Scale bars=100 *μ*m. (**f**) Kaplan–Meier curve showed that patients with high 5-hmC levels had longer OS and lower tumor recurrence possibility

High 5-hmC levels were detected by immunofluorescence (IF) staining in the peritumoral liver tissues, and the 5-hmC signal was absent in corresponding tumor tissues (Supplementary Figure S1A). Using 5-hmC immunohistochemical (IHC) staining, we found that normal and peritumoral liver tissues showed strong nuclear 5-hmC staining, whereas virtually all HCC tumor cells exhibited partial or complete 5-hmC loss (Figures 1a–c). Next, we confirmed higher 5-hmC levels in normal and peritumoral tissues than in HCC tissues using anti-5-hmC dot blot assay (Figure 1d). Moreover, we examined 5-hmC levels by IHC staining a tissue microarray (TMA) comprised of 323 HCC paraffin-embedded tissues. Consistent with the individual cases (Figures 1b and c), the TMA confirmed significant 5-hmC loss in tumor tissues compared with corresponding peritumoral liver tissues (Figure 1e and Supplementary Figure S1B). We then determined whether 5-hmC levels were correlated with critical HCC tumor, node, and metastases (TNM) stage. The 5-hmC staining score negatively correlated with TNM stage. Specifically, 5-hmC levels in TNM stage I HCC were significantly higher than in TNM stage II–III HCC (Supplementary Table S1).

We analyzed the association between 5-hmC levels and survival probability based on data from all 323 patients. The Kaplan–Meier curves revealed that patients with high 5-hmC levels (staining score ⩾2) had significantly higher survival probabilities than patients with low 5-hmC levels (staining score <2). High 5-hmC levels were associated with better overall survival (OS). The overall 1-, 3-, and 5-year OS rates in the low 5-hmC group were 80.5, 55.9, and 45.1%, respectively. These OS rates were significantly lower than the high 5-hmC group (89.1, 69.5, and 57%, respectively; Figure 1f). In addition, low 5-hmC levels were significantly correlated with early HCC recurrence following hepatic resection. The median recurrence-free survival (RFS) was substantially reduced among low 5-hmC levels patients (28 months *versus* 56 months, *P*<0.01). The 1-, 3-, and 5-year RFS rates were significantly higher in the high 5-hmC group (75.8, 55.9, and 47.5%, respectively) than the low 5-hmC group (69.8, 41.5, and 35.1%, respectively; Figure 1f). Moreover, 5-hmC loss significantly correlated with shorter OS (*P*=0.011, hazard ratio [HR]=0.654) and time to recurrence (TTR; *P*=0.004, HR=0.634; Table 1). Taken together, these data indicated that 5-hmC expression was an independent factor for HCC prognosis.

### 5-hmC decrease is associated with substantial TET-family expression reduction in HCC

**Figure 2:**
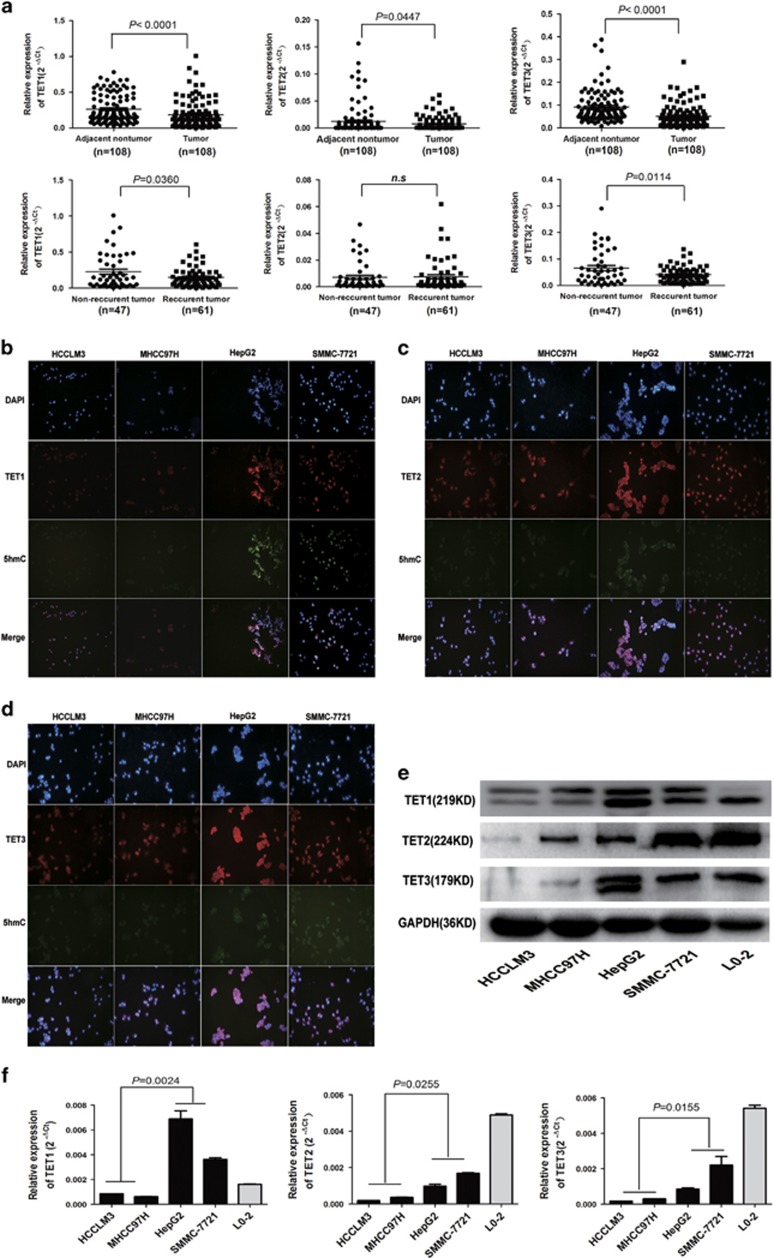
Downregulation of TET-family members is associated with decreased 5-hmC levels in HCC. (**a**) Relative TET1, 2, and 3 levels among peritumoral and tumoral tissues using qRT-PCR, The patients suffering HCC recurrence exhibited lower mRNA TET1 and 3 levels compared to patients without recurrence, respectively. (**b**–**d**) Immunofluorescence staining analysis TETs and 5-hmC levels in different HCC cell lines. (**e** and **f**) Relative TET1, 2, and 3 mRNA and protein levels in different HCC cell lines and normal liver cell line L-02. Data are shown as mean±S.D. (*n*=3)

Next, we investigated cellular factors responsible for 5-hmC loss in HCC. While the TET family is directly responsible for 5-hmC generation, the co-factor *α*-ketoglutarate (*α*-KG) is absolutely required 5-mC to 5-hmC conversion,^6^ *α*-KG is mainly produced by isocitrate dehydrogenases (IDHs).^17^ To determine whether IDH and /or TET-family enzymes are responsible for 5-hmC loss in HCC, we examined IDH and TET-family genes expressions in HCC tumor tissues (cohort 1, *n*=108) using quantitative real-time polymerase chain reaction (qRT-PCR). Strikingly, the expression of all three TET genes were significantly lower in tumor than in adjacent peritumoral liver tissues (all *P*<0.05, Figure 2a). IDH1 and IDH2 expressions were similar in HCC and peritumoral liver tissues (Supplementary Figure S1C). Furthermore, TET1 and TET3 expressions were decreased in tumor tissues from patients with intrahepatic tumor recurrence or extrahepatic metastasis compared with patients with no tumor recurrence (both *P*<0.05, Figure 2a). These findings were corroborated at the protein levels using IHC staining (Supplementary Figure S1D).

To further evaluate TET-family enzymes and 5-hmC’s association with HCC metastasis, we analyzed TET enzymes and 5-hmC levels in a panel of human HCC cell lines with different metastatic potentials (Figures 2b–f). Higher TET1, 2, 3 and 5-hmC expressions were detected in low metastatic HCC cells lines (SMMC-7721 and HepG2), whereas lower TET1, 2, 3 and 5-hmC expressions were detected in highly metastatic HCC cell lines HCCLM3 and MHCC97H. Taken together, these data suggest that diminished TET-family expression represents a molecular mechanism underlying global 5-hmC loss in HCC.

### miR-29a regulates 5-hmC level by directly targeting TET-family members in HCC cells

**Figure 3:**
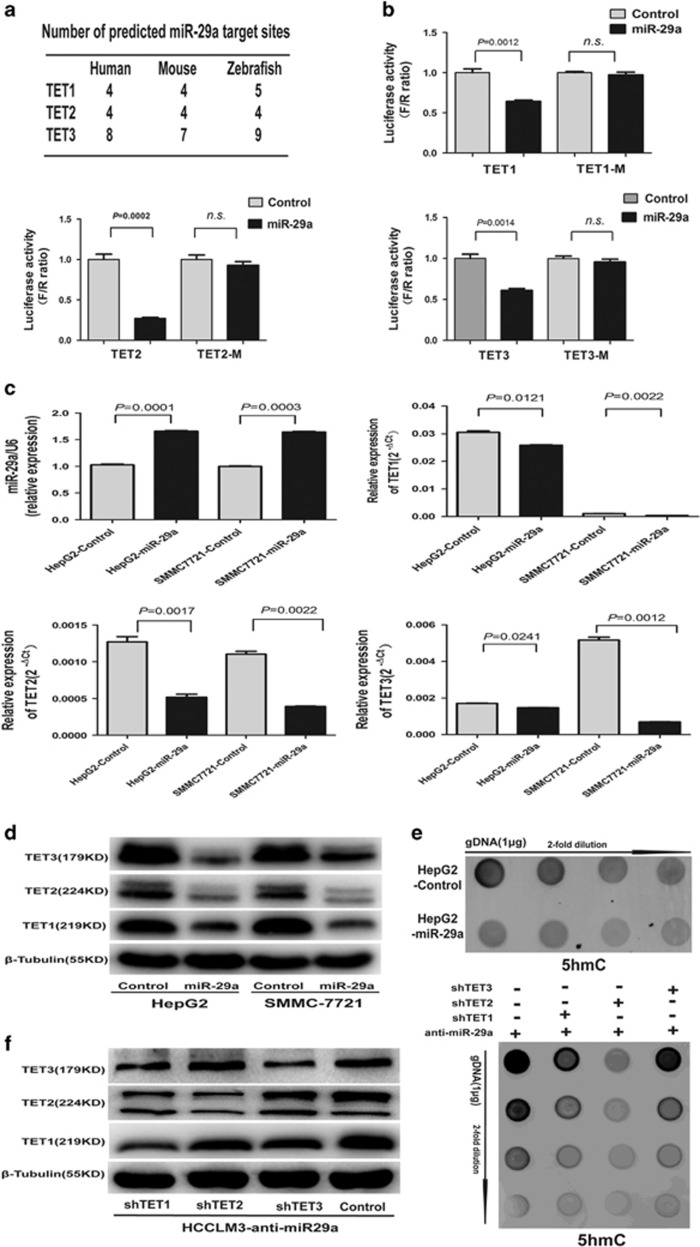
miR-29a decreased 5-hmC levels by directly targeting TET methylcytosine dioxygenases. (**a**) miR-29a target sites predicted by TargetScan for human, mouse, and zebrafish TET-family members. (**b**) miR-29a significantly suppressed the luciferase activity of TET family containing a wild-type 3'-UTR, but showed no effect on the activity of TET family with a mutant 3'-UTR. (**c** and **d**) Following transfection with miR-29a vector, qRT-PCR and western blotting experiments showed the mRNA and protein levels of TET1, 2, and 3 in SMMC-7721 and HepG2 cells were significantly decreased compared with controls. (**e**) Global 5-hmC levels in HepG2-miR-29a cells and control miRNA were measured. (**f**) shTET1, shTET2, and shTET3 in HCCLM3-anti-miR-29a cells were determined (left). Global 5-hmC levels in shTET1, 2, and 3 and negative control groups were measured (right). Data are shown as mean±S.D. (*n*=3)

Recent studies demonstrated that miRNAs (e.g., miR-22), which may contribute to global epigenetic alterations, directly target TET proteins.^16, 18^ We utilized prediction algorithms, including TargetScan 6.0 (http://targetscan.org)^19^ and miRBase (http://www.mirbase.org)^20^ to search for miRNAs that potentially regulate TET enzymes in HCC and identified three miRNAs, miR-22, miR-26a, and miR-29a. As miR-29a ranks the highest target score among them, we focused on miR-29a. The miR-29 family have at least three putative target binding sites in TET1, 2 and 3 in the human, mouse genomes and zebrafish, suggesting strong conservation (Figure 3a). Notably, miR-29a target sites are preferentially located near all ends of the TET family members 3′-UTR (Supplementary Figures S2A and B), implying effective targeting. Moreover, miR-29a coexpression effectively downregulated luciferase expression in the TET family members 3′-UTRs constructs. Seed sequence mutations of the predicted miR-29a binding sites within TET family members abolished miR-29a’s inhibitory effects on luciferase expression (Figure 3b; Supplementary Figure S2A). Consistent with previous reports,^21^ these results firmly confirmed a direct interaction between miR-29a and the 3′-UTR region of TET genes.

To further evaluate miR-29a’s association with HCC metastasis, we analyzed miR-29a levels in a panel of human HCC cell lines with different metastatic potentials.^22^ The miR-29a expressions in highly metastatic HCC cell lines HCCLM3 and MHCC97H were considerably higher than in low metastatic potential SMMC-7721 and HepG2 (Supplementary Figure S2C). Moreover, miR-29a upregulation in SMMC-7721 and HepG2 cells led to robust TET enzymes downregulation and global 5-hmC levels reduction (Figures 3c–e). In contrast, miR-29a inhibition in HCCLM3 and MHCC97H cells resulted in significant TET enzymes expression elevation and a global 5-hmC level increase (Supplementary Figures S3A–C). Furthermore, RNA interference (RNAi) downregulation of TET1, 2, and 3 in miR-29a-inhibited HCCLM3 cells reduced global 5-hmC levels (Figure 3f). Taken together, these data suggest that 5-hmC epigenetic marker changes may contribute to miR-29a’s impact on HCC progression and metastasis by targeting TET-family members.

### miR-29a promotes HCC cells proliferation and invasion by directly targeting TET-family members

**Figure 4:**
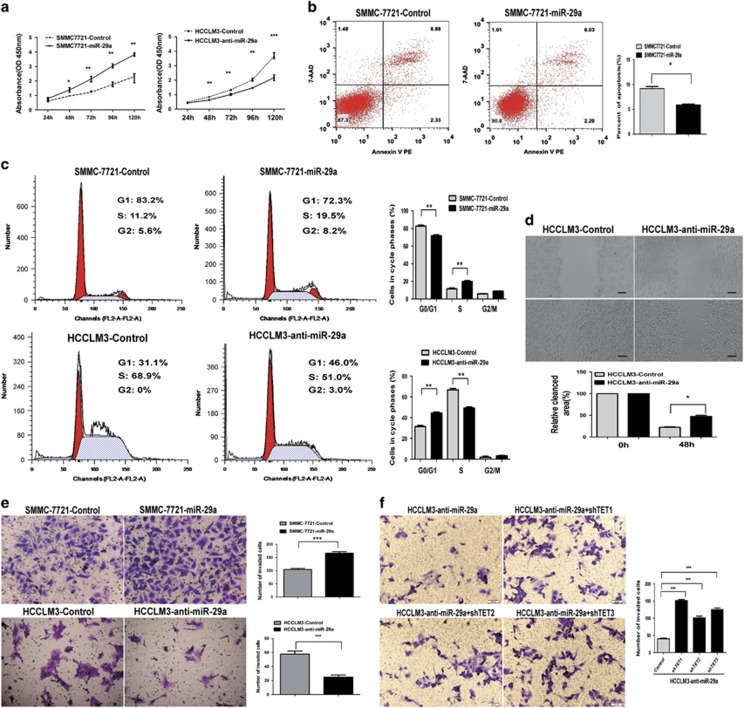
The effects of miR-29a on HCC cells proliferation, apoptosis, migration, and invasion via directly targeting TET-family protein *in vitro*. (**a**) Cell proliferation of miR-29a group was significantly enhanced compared with controls, whereas miR-29a inhibited caused the opposite result. (**b**) miR-29a upregulation significantly decreased apoptosis rate compared with controls. (**c**) Cell-cycle analysis showed that miR-29a inhibitors caused S-phase arrest (S-phase, 50.8±1.9 *versus* 70.6±1.6%, respectively), whereas miR-29a overexpression caused the opposite result. (**d**) miR-29a knockdown significantly decreased wound healing compared with control cells. (**e**) The invaded cells number in SMMC-7721-miR-29a cells significantly increased compared with control cells, whereas miR-29a inhibitors decreased them. (**f**) TET1, TET2, and TET3 knockdown in HCCLM3-anti-miR-29a cells enhanced cells invasion compared with control cells. Data are shown as mean±S.D. (*n*=3). *P<0.05, **P<0.01, and ***P<0.001. Scale bars=100 *μ*m

To further explore miR-29a’s biological significance in HCC, we transfected miR-29a expression vectors and anti-miR-29a vectors into human HCC cell lines. miR-29a expression was verified by qRT-PCR (Supplementary Figure S3A). miR-29a upregulation in SMMC-7721 cells significantly promoted cell proliferation and inhibited apoptosis (Figures 4a and b). In addition, miR-29a knockdown in HCCLM3 cells significantly suppressed cell proliferation and induced apoptosis compared to control cells (Figure 4a; Supplementary Figure S4A). Cell-cycle analysis revealed that miR-29a inhibition caused an S-phase arrest (S-phase, 50.8±1.9 *versus* 70.6±1.6%, respectively), whereas miR-29a overexpression caused the opposite results (Figure 4c).

In addition, SMMC-7721 cells mobility in wound healing assays significantly increased with miR-29a overexpression (Supplementary Figure S4B). In contrast, miR-29a knockdown decreased wound healing in HCCLM3 cells (Figure 4d). Similarly, miR-29a upregulation significantly increased SMMC-7721 cells invasion, whereas miR-29a silencing markedly decreased HCCLM3 cells invasion (*P*<0.01, Figure 4e). We knocked down TET1, TET2, and TET3 using RNAi in HCCLM3-anti-miR-29a cells, the dramatic cell invasion reduction induced by anti-miR-29a was reversed by TET proteins knockdown (Figure 4f). Taken together, these data suggest that miR-29a significantly enhances HCC cells proliferation and invasion, TET-family members are the major players responsible for miR-29a’s functions.

### miR-29a triggers methylation-dependent SOCS1 silencing and promotes HCC tumor growth and metastasis *in vivo*

**Figure 5:**
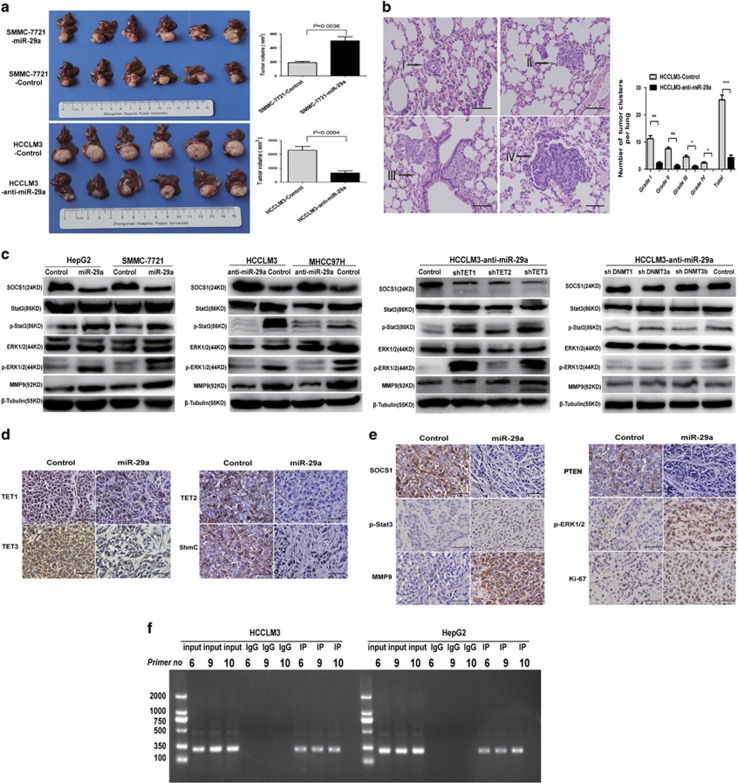
miR-29a enhances HCC tumor growth and metastasis *in vivo* by inhibiting the TET–SOCS1–MMP9 signaling pathway. (**a**) miR-29a overexpression promoted tumor growth in orthotopic SMMC-7721 cells implantation mouse models, whereas miR-29a inhibited caused the opposite result (left). Tumor volumes at week 6 were quantitated (right). (**b**) Metastatic lesions in the lungs of mice at week 6 (left), the total numbers and grades of lung metastatic lesions in the different groups of nude mice were measured (right). Data are shown as mean±S.D. (*n*=3). *P<0.05, **P<0.01, and ***P<0.001. (**c**) In SMMC-7721 and HepG2 cells transfected with miR-29a vector, expression levels of p-Stat3, p-ERK1/2, and MMP9 were significantly increased compared with control cells. While miR-29a inhibited caused the opposite result. TET1, 2, and 3 knockdown in HCCLM3-anti-miR-29a cells promoted p-Stat3, p-ERK1/2, and MMP9 expressions. DNMT1 and DNMT3A knockdown in HCCLM3-anti-miR-29a cells, which had no significant effect on SOCS1, p-Stat3, p-ERK1/2, and MMP9 expressions, downregulation DNMT3B slightly decreased p-Stat3 and p-ERK1/2 expressions. (**d** and **e**) IHC staining for TET–SOCS1–MMP9 pathway-related proteins in tumor tissues from orthotopic HCC implantation mice. Scale bars=100 *μ*m. (**f**) ChIP-PCR assays demonstrated the binding of TET1 to the SOCS1 promoter in HCC cells

To elucidate miR-29a’s tumor growth and metastasis effects *in vivo*, we orthotopically transplanted the various HCC cell lines into nude mice and assessed the resultant liver tumors. The average tumor size of SMMC-7721-miR-29a-derived xenografts was 502±144.4 mm^3^, markedly larger than SMMC-7721-control-derived tumors (190±43.4 mm^3^, *P*=0.0036; Figure 5a). Pulmonary metastasis occurred in 66.7% (4/6) of SMMC-7721-miR-29a mice *versus* 0% (0/6) in SMMC-7721-control mice. The average tumor size of HCCLM3-control-derived xenografts (2291±681 mm^3^) were significantly larger than HCCLM3-anti-miR-29a-derived xenografts (662±376.1 mm^3^, *P*=0.0004; Figure 5a). The lung metastasis incidence of orthotopic HCCLM3-anti-miR-29a-derived tumors was 16.7% (1/6), whereas this incidence was 100% (6/6) in HCCLM3-control group. The total number and grade of lung metastatic lesions in HCCLM3-anti-miR-29a group was much lower than HCCLM3-control group (*P*<0.01, Figure 5b). Taken together, these results indicate that miR-29a has a crucial role in HCC proliferation, invasion, and metastasis *in vivo*.

Recent studies showed that CpG island hypermethylation-mediated epigenetic silencing of tumor suppressor genes including APC, RASSF1A, SOCS1, HIC1, GSTP1, CDKN2A, RUNX3, and PRDM2 were involved in HCC.^3, 4^ Notably, SOCS1 ranked first among the altered tumor suppressor genes in HCC cells transfected with miR-29a mimics and inhibitors (Supplementary Figure S5A). We hypothesized that miR-29a antagonizes SOCS1 transcription by controlling genomic 5-hmC levels. We analyzed the SOCS1 promoter using glucMS-qPCR and found that it is hypermethylated upon miR-29a overexpression (Supplementary Figure S5B). Indeed, miR-29a overexpression significantly inhibited SOCS1 expression at both RNA and protein levels (Figure 5c and Supplementary Figure S5A). Furthermore, treating these cells with the DNA-demethylating agent, 5′-Aza, reversed miR-29a’s effects and restored SOCS1 expression transcripts (Supplementary Figure S5C).

Promoter hypermethylation and subsequent inactivation of SOCS1 is associated with JAK/STAT3 pathway activation, which contributes to HCC development and progression.^3, 4, 23^ We examined several SOCS1–JAK/STAT3 pathway cognate target genes, including SOCS1, p-Stat3, and MMP9 using western blot assays. p-Stat3, p-ERK1/2, and MMP9 expressions were increased in SMMC-7721 and HepG2 cells stably overexpressing miR-29a (Figure 5c). In contrast, the expressions of these genes were significantly downregulated in HCCLM3 and MHCC97H cells treatment with miR-29a inhibitors (Figure 5c). Furthermore, TET1, 2, and 3 downregulation via RNAi in HCCLM3-anti-miR-29a cells affected SOCS1 and its target genes expressions, reversed the anti-miR-29a effects alone (Figure 5c). Similar to our cell lines studies, the intensities for TET1, 2, 3, 5-hmC, and SOCS1 were significantly decreased, whereas p-Stat3, p-ERK/2, and MMP9 were greatly increased in SMMC-7721-miR-29a-derived xenograft tissues compared with controls (Figures 5d and e).

The above data suggest that TET-family enzymes regulates SOCS1 expression in HCC cells. Therefore, we assessed the possibility of TETs-dependent regulation of SOCS1 mRNA transcription. Using ChIP-PCR analyses, our results indicated TET1 protein bind to the promoter of SOCS1 in HCC cells. We also found that TET1 can physically bind to multiple common binding sites (#6, #9, and #10) on the SOCS1 promoter in HCCLM3 and HepG2 cell lines (Figure 5f). These findings suggest that TET1 is a critical regulator of SOCS1 gene expression, and specifically, multiple binding sites are essential for TET1-induced SOCS1 transactivation.

In addition, recent studies indicated that miR-29a specifically targets both DNA methyltransferases (DNMT3A and DNMT3B) in cancer.^24, 25^ The findings of our current study showed that a significant DNMT1, DNMT3A, and DNMT3B protein expressions increased in highly invasive HCCLM3 and MHCC97H cells compared with SMMC-7721 and HepG2 cells (Supplementary Figure S6A). The protein levels of DNMT3A and DNMT3B in SMMC-7721 and HepG2 cells transfected with miR-29a vector, were significantly decreased compared with controls (Supplementary Figure S6B). Then, DNMT3A and DNMT3B knockdown in HCCLM3-anti-miR-29a cells, we observed that 5-hmC levels and HCC cells invasive behavior had no significant alteration. DNMT3B knockdown in HCCLM3-anti-miR-29a cells promoted HCC cells apoptosis (Supplementary Figures S6C–E). In addition, DNMT1, DNMT3A, and DNMT3B knockdown in HCCLM3-anti-miR-29a cells had no significant effect on SOCS1, p-ERK1/2, and MMP9 expressions, except that downregulation DNMT3B slightly decreased p-Stat3 and p-ERK1/2 expressions (Figure 5c). Overall, these findings revealed that miR-29a regulates 5-hmC levels in HCC by directly targeting TET proteins, rather than DNMT3A and DNMT3B. Importantly, these data confirmed that miR-29a suppresses SOCS1 gene promoter demethylation and represses its expression, leading to cognate targets upregulation to promote tumor growth and metastasis.

### miR-29a overexpression correlates with poor clinical outcomes and TET–SOCS1–MMP9 axis silencing in HCC patients

**Figure 6:**
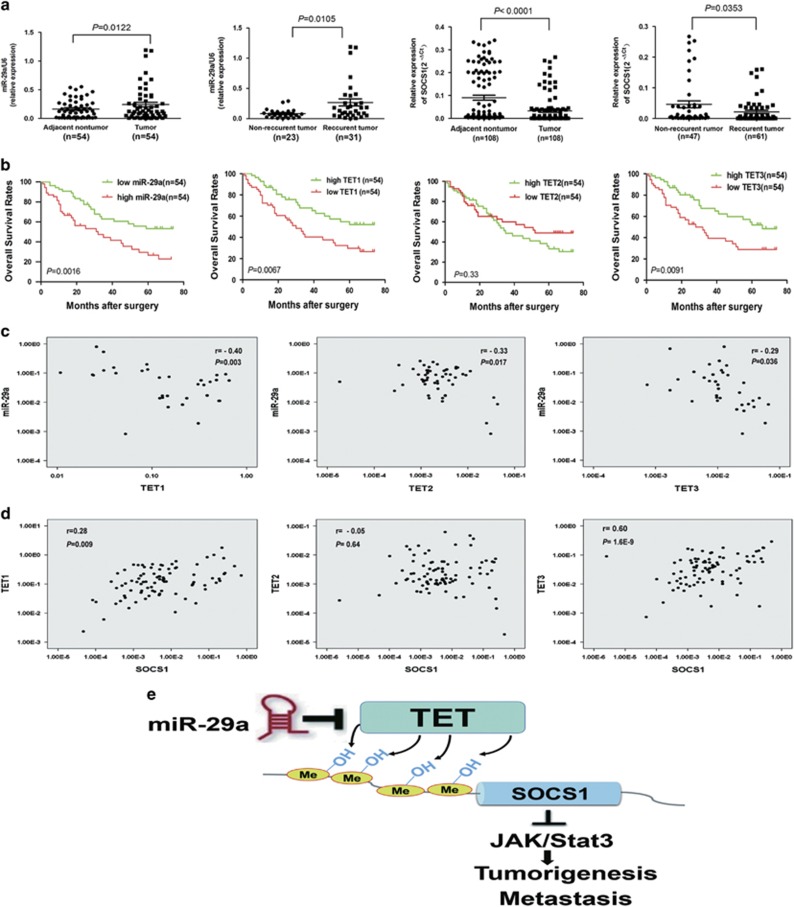
miR-29a overexpression correlates with poor clinical outcomes and TET–SOCS1–MMP9 axis silencing in HCC patients. (**a**) Relative expressions of miR-29a and SOCS1 among HCC and adjacent non-tumor tissues. Data are shown as mean±S.D. (*n*=3). (**b**) Kaplan–Meier curve showed that patients with high TET1 or, TET3 levels had longer OS, whereas those with high miR-29a level had shorter OS. (**c**) The correlation between miR-29a and TET-family expressions was analyzed. (**d**) Correlation analysis of each TET family with SOCS1 was analyzed. (**e**) Proposed model for miR-29a’s role in proliferation and metastasis promotion through epigenetic SOCS1 inactivation via direct TET-family targeting. miR-29a decreases 5-hmC level by negatively regulating TET-family member expression, resulting in epigenetic SOCS1 inactivation due to reduced 5-hmC levels. Ultimately, SOCS1 dysfunction triggers activation STAT3 signaling, which leads to MMP9 expression upregulation and increased tumor invasiveness and metastasis

Finally, we assessed the association between miR-29a overexpression and HCC patient clinical outcomes. We analyzed miR-29a expression by qRT-PCR in 54 tumor tissues and showed that miR-29a expressions were significantly upregulated in HCC tissues compared to peritumoral liver tissues (*P*<0.05, Figure 6a). Furthermore, miR-29a levels were increased in tumor tissues from patients with intrahepatic tumor recurrence or extrahepatic metastasis compared with recurrence-free patients (*P*<0.05, Figure 6a). Critically, HCC patients with high miR-29a expressions had poor survival rates as compared with low miR-29a expression patients (*P*=0.0016, Figure 6b). These results indicate that significant miR-29a expression upregulation occurs in HCC and correlates with HCC relapse and metastasis.

Interestingly, we found that SOCS1 expression significantly decreased in tumor tissues compared with corresponding adjacent tumor tissues and correlated with early recurrence (*P*<0.05, Figure 6a). The HCC patients with high TET1 or TET3 expression levels harbored higher OS rates compared with low TET1 or TET3 expression patients (both *P*<0.01, Figure 6b). However, the high TET2 expression patients did not have significantly different OS rates than low TET2 expression patients (Figure 6b). More importantly, and consistent with our functional studies, miR-29a expression was negatively correlated with TET1, TET2, and TET3 expression in HCC patients (*n*=54, *r* =−0.4, −0.33, and −0.29, respectively; all *P*<0.05; Figure 6c). However, there was a weak inverse correlation between miR-29a and DNMT3A expression in HCC patients (*r*=−0.165, *P*=0.047; Supplementary Figure S6F). Lastly, we examined whether TET-family downregulation by miR-29a explains its effects on SOCS1 expression in HCC tissues. We found a clear correlation between TET1 and TET3 expression and SOCS1 in HCC patients (*n*=108, *r*=0.28 and 0.60, respectively, both *P*<0.01; Figure 6d). Taken together, these data support the notion that miR-29a is a crucial epigenetic modifier and promoter of HCC metastasis via TET family–SOCS1 pathway repression.

## Discussion

HCC is one of the most lethal tumors, and few effective therapeutic options are available. Understanding hepatocarcinogenesis molecular mechanisms could provide strategies to arrest, retard, or even reverse the disease in HCC patients.^26^ Several recent studies suggested that 5-hmC contents were reduced in solid tumors, including breast, pancreas, liver, and lung tumors.^9, 10, 27, 28^ In this study, we found that 5-hmC loss was a distinctive epigenetic event in HCC progression, and that it correlated with shorter OS and RFS, suggesting that 5-hmC loss could potentially be a molecular biomarker with predictive and prognostic value. Taken together, these data suggested that 5-hmC may represent an epigenetic marker for HCC identification and progression.

Two mechanisms have been reported that dysfunction of IDH and**/**or TET enzymes, two key factors in 5-hmC generation, that would lead to greatly decreased 5-hmC levels in human tumors: loss-of-function mutations target TET2 and IDH1/2 mutations that result in TET activity inhibition via *α*-KG reduction and 2-hydroxyglutarate accumulation.^17, 29, 30^ HCC harbors no IDH1 mutations,^31^ recent studies found IDH2 protein decreased in HCC.^9^ Herein, we demonstrated IDH1 and IDH2 had similar RNA expressions in both HCC and peritumoral liver tissues. Remarkably, a significant TET1, TET2, and TET3 gene expression decrease was detected in HCC compared with peritumoral liver tissues, suggesting that insufficient TET enzymes may be one mechanism underlying global 5-hmC loss in HCC.

miR-29a has previously been shown to have an effect on the development HCC but the findings have been contradictory.^24, 32, 33^ Two studies supported miR-29 miRNAs directly regulate expression of TET protein in noncancer cells and HCC.^21, 34^ However, another report revealed that AFP expression transcriptionally regulated miR-29a and upregulated miR-29a suppresses the proliferation of HCC cells by targeting DNMT3A.^24^ This observation, which contradicts our study results, may stem from the difference cell lines HLE cells are negative for AFP. While HCC cell lines used in our study all endogenously express AFP. Our findings indicated miR-29a expressions were positively correlated with the metastatic potential of HCC cells. Upregulation of miR-29a also predicted tumor metastasis and poor prognosis for HCC patients, suggesting a tumor-promoting role for this miRNA. We also revealed that miR-29a upregulation significantly reduced TET family members expressions in HCC cells. Conversely, miR-29a downregulation increased TET family members expressions. Moreover, miR-29a overexpression decreased luciferase reporter activity in a wild-type TET family 3′-UTR but not the mutant 3′-UTR. miR-29a upregulation and downregulation in HCC cells yielded distinct global 5-hmC expressions, and the loss of 5-hmC was associated with miR-29a overexpression in HCC cells. More importantly, TET protein inhibition triggers a proliferation and invasion phenotype similar to that induced by miR-29a overexpression in HCC cells. We validated the inverse correlation between miR-29a mRNA levels and TET1, 2, and 3 expression in HCC tissues. Overall, these data demonstrated that TET protein served as a major miR-29a target for HCC progression modulation.

SOCS1 silencing by CpG island hypermethylation in HCC has been demonstrated in several previous studies.^3, 23^ In addition, due to SOCS1’s role as a negative JAK/STAT pathway regulator, it may also be an HCC tumor suppressor.^23^ Our findings indicated that miR-29a suppressed SOCS1 mRNA expression by directly targeting TET enzymes, leading to SOCS1 promoter hypermethylation, SOCS1 suppression and STAT3 signaling activation. These effects resulted in MMP9 expression upregulation and carcinogenesis induction, increasing tumor invasiveness and metastasis (Figure 6e). Importantly, we observed that TET1 protein bind to the promoter of SOCS1 in HCC cells by ChIP-PCR analyses, and three common binding sites of SOCS1 were found on the promoter in HCCLM3 and HepG2 cell lines. Indeed, aberrant SOCS1 CpG islands DNA methylation appears to be closely linked to their silencing during HCC metastasis, whereas epigenetic modifier drugs such as 5′-Aza can reactivate SOCS1 expression, indicating that epigenetic mechanisms have a functional role in controlling expression of this factor in cancers.^4, 23^ Therefore, miR-29a induces TET protein suppression leads to decreased expression of SOCS1 in HCC, this might be one mechanism for miR-29a promote HCC invasion and metastasis.

In addition, recent studies indicated that miR-29a specifically targets both DNMT3A and DNMT3B in HCC.^24, 25^ Silenced DNMT3A and DNMT3B protein expression using siRNA, we observed that 5-hmC levels and HCC cells invasive behavior had no significant alteration. Indeed, the absence of DNMT1 and DNMT3A had no significant effect on SOCS1, p-Stat3, p-ERK1/2, and MMP9 expressions compared with control vector, except slightly decreased p-Stat3 and p-ERK1/2 in downregulation DNMT3B cells. In this study, our findings revealed that miR-29a regulates 5-hmC levels in HCC by directly targeting TET protein, rather than DNMT3A and DNMT3B. A recent report proposed miR-29a regulated the Yin-yang dynamics of DNA methylation in disease and development by targeting both TET family members and DNMTs.^35^ These data suggest miR-29a dysregulation in cancer induces a phenotype of DNA methylation instability that could facilitate tumorigenesis.^25^

These findings provide important insights into the molecular basis of tumor metastasis but also have important implications for cancer diagnosis, prognosis, and treatment. As miR-29a triggers a metastatic phenotype and its overexpression correlates with poor clinical outcomes in HCC patients, miR-29a might be a useful biomarker to identify metastatic forms of HCC. Our findings suggest that miR-29a inhibition via specific miR-29a inhibitors in highly metastatic cells reduces the metastatic phenotypes as well as elevating TET family members and SOCS1 expression provide a solid rationale for therapeutic miR-29a targeting to prevent HCC metastatic phenotypes.

In conclusion, our findings revealed that miR-29a decreases 5-hmC levels by negatively regulating TET-family expression and the subsequent epigenetic SOCS1 inactivation. Ultimately, SOCS1 dysfunction triggers MMP9 upregulation, which increases HCC cell proliferation, invasion, and metastasis. Therefore, miR-29a modulates HCC progression through the TET–SOCS1–MMP9 axis, which may present a useful clinical target for therapeutic interference of HCC progression.

## Materials and methods

### Cell lines and animals

HEK293T, four human HCC cell lines with differing metastatic potentials (HepG2, SMMC-7721, MHCC97H, and HCCLM3), and one human non-transformed hepatic cell line L-02 were used in this study. HCC cell lines with stepwise metastatic potential (MHCC97H and HCCLM3, which have the same genetic background but different lung metastatic potentials) were established at our institute,^36, 37^ and authenticated by short tandem repeat validation analysis during the study period. The HCC cell lines HepG2 and SMMC-7721 and the normal liver cell line L-02 were purchased from the cell bank of Chinese Academy of Sciences (Shanghai, China). These cell lines were obtained within 6 months before being used in this study. All cell lines were maintained in high glucose Dulbecco's modified Eagle medium, supplemented with 10% FBS and penicillin/streptomycin, at 37 °C under 5% CO_2_ in incubator.

Male BALB/c nu/nu mice (4–6 weeks old, Shanghai Institute of Material Medicine, Chinese Academy of Science) were raised in specific pathogen-free conditions. All procedures were performed in accordance with the National Institute of Health Guide for the Care and Use of Laboratory Animals.

### Patients and tissue samples

Two independent cohorts comprised of 431 HCC patients were enrolled in this study. The 108 tumor samples and matched non-tumor liver tissues (cohort 1, snap-frozen tissues) were consecutively collected from patients undergoing curative resection in 2007 between January and December at our institute. The other cohort (cohort 2, *n*=323, paraffin-embedded tissues) was randomly collected from patients with HCC undergoing curative resection from 2003 to 2004. Ethical approval for the use of human subjects (No. 2009-002) was obtained from the Research Ethics Committee of Zhongshan Hospital (Shanghai, China), and informed consent was obtained from each patient. Detailed information and follow-up procedures are described in the Supplementary Materials and Methods.

### Transduction of lentiviral vectors

Four HCC cell lines (HepG2, SMMC-7721, MHCC97H, and HCCLM3) were transfected using lentiviral vectors (miR-29a expression vector, the control vector for miR-29a, miR-29a inhibitor, and the negative control for the miR-29a inhibitor) according to the manufacture’s instruction (GeneChem, Shanghai, China). The detailed protocols are shown in Supplementary Materials and Methods.

### TMA and immunohistochemistry assay

TMAs were constructed as previously described.^38^ Two core biopsies of 1 mm in diameter were taken from the donor blocks and transferred to the recipient paraffin block at defined array positions. The TMA was constructed from the 323 cases in cohort 2. IHC staining was performed by the avidin–biotin–peroxidase complex method. Briefly, after rehydration and microwave antigen retrieval, polyclonal antibodies against 5-hmC (1:500, Catalog No. 39769, Active Motif, Carlsbad, CA, USA) were applied to slides, incubated at 4 °C overnight. Then slides were incubated with secondary antibody (GK500705, Gene Tech, Shanghai, China) at 37 °C for 30 min. Staining was carried out with 3,3′-diaminobenzidine (DAB), and Mayer’s hematoxylin was used for counterstaining. Negative control slides with the primary antibodies omitted were included in all assays. The scoring system are shown in Supplementary Materials and Methods.

### RNA isolation and qRT-PCR

Total RNA was extracted from cell lines and frozen tumor specimens using the Trizol reagent (Invitrogen, Carlsbad, CA, USA). Complementary DNA synthesis was performed using a High Capacity cDNA Reverse Transcription kit or TaqMan MicroRNA Reverse Transcription kit (Applied Biosystems, Life Technologies, Carlsbad, CA, USA) according to the manufacturer’s instructions. The mRNA or miRNA expression in HCC cell lines, 108 HCC samples, and adjacent normal liver tissues were measured by RT-qPCR using an ABI7900HT instrument (Applied Biosystems). Levels of TET1, TET2, TET3, SOCS1, IDH1, IDH2, and miR-29a were normalized to GAPDH or U6. All experiments were performed in triplicate. The Taqman 2112 and 1973 probes were used for detection for miR-29a and U6, respectively (Applied Biosystems). The primers used in qRT-PCR assay are listed in Supplementary Table S2.

### Western blot analysis

Western blotting was performed as previously described.^39^ Briefly, total cell lysates were generated and proteins were separated on a 10% SDS-PAGE, followed by transfer to polyvinylidene difluoride membranes. The membranes were washed and blocked. The primary antibodies and dilutions used were as follows: p-ERK1/2 (Thr202/Tyr204), ERK1/2, p-STAT3 (Tyr705), and STAT3 (1:1000, Cell Signaling Technology, Beverly, MA, USA); TET1 (1:1000, clone ab191698, Abcam, Cambridge, MA, USA); TET2 (1:800, clone ab94580, Abcam); TET3 (1:1000, clone ab139805, Abcam); SOCS1(1:800, clone ab83493, Abcam); PTEN (1:800, clone ab31392, abcam); MMP9 (1:1000, clone ab38898, Abcam); tubulin (1:1000; clone 2A9, Abnova, Taipei, Taiwan); and GAPDH (1:5000; Millipore, Bedford, MA, USA). Samples were incubated with primary antibodies then with horseradish peroxidase- conjugated secondary antibodies. Antibody binding was detected by enhanced chemiluminescence assays.

### Immunofluorescence staining

For IF assays, cells cultured on glass slides were fixed in 4% paraformaldehyde for 15 min. Subsequently, the cells were permeabilized with 0.1% Triton X-100 for 15 min at room temperature, washed with phosphate buffered saline (PBS), and blocked with PBS containing 5% (w/v) bovine serum albumin (BSA) and 0.15% (w/ v) glycine (BSA buffer) for 1 h at room temperature. Cells were treated with anti-TET1 (1:300, clone ab191698, Abcam), TET2 (1:250, clone ab94580, Abcam), TET3 (1:300, clone ab139805, Abcam), and 5-hmC (1:400, clone ab106918, Abcam) antibody for 24 h at 4 °C. A negative control with primary antibody omitted was included on every slide. Cells were then washed with BSA buffer and incubated with 2 *μ*g/ml Alexa Fluor 488-conjugated goat anti-rat antibody and 1 *μ*g/ml Alexa Fluor 647-conjugated goat anti-rabbit antibody (Abcam) for 1 h at room temperature. After rinsing in PBS, the slides were counterstained with diamidino phenylindole and examined by fluorescence microscopy (Leica Microsystems Imaging Solutions, Cambridge, UK).

### Luciferase reporter assay

HEK293T cells were seeded in a 96-well plate at 50–60% confluence. After 24 h, cells were transfected with 120 ng of miR-29a mimics or a negative control. Cells were cotransfected with 30 ng of modified pGL3 plasmids containing the wild-type 3′-UTR of TET1, 2, and 3 or a mutant 3′-UTR of TET1, 2, and 3 with all putative target sequences mutated. Transfections were performed using 0.45 *μ*l of Fugene (Promega, Madison, WI, USA). Cells were collected 48 h after transfection, and Renilla luciferase activity was measured using a dual-luciferase reporter system (Promega). Luciferase reporter assays were performed in duplicate and repeated in three independent experiments. Luciferase activity was detected using an Orion II microplate luminometer (Berthold Technologies, Bad Wildbad, Stuttgart, Germany).

### Chromatin immunoprecipitation analysis

Chromatin immunoprecipitation assay was performed as described previously.^40^ Quantification of precipitated DNA by chromatin immunoprecipitation was performed using RT-PCR amplification. Experimental details are described in the Supplementary Materials. The primers used in the amplification are listed in Supplementary Table S3.

### *In vivo* assays for tumor growth and metastasis

SMMC-7721-miR-29a, SMMC-7721-control, HCCLM3-anti-miR-29a, and HCCLM3-control cells (5 × 10^6^) were suspended in 100 *μ*l serum-free Dulbecco’s modified Eagle medium and Matrigel (BD Biosciences, Franklin Lakes, NJ, USA) at a (1:1 ratio) and then injected subcutaneously into the upper left flank region of nude mice. Liver orthotopic transplantation model were also performed according standard procedures described previously.^41^ At the end of study, mice were killed and tumor tissues were collected, photographed and the volume of tumors was calculated in mm^3^ as follows: *V*=*ab*^2^/2 (with a and b representing the largest and smallest tumor diameters measured at necropsy, respectively).^42^ The detailed protocols are shown in Supplementary Materials and Methods.

### Dot blot

Genomic DNA was isolated from samples with a QIAamp mini DNA kit (Qiagen, Duesseldorf, Germany) according to the manufacturer’s instructions. Genomic DNA was denatured with 0.1 M NaOH and spotted on nylon membranes (Millipore, Bedford, MA, USA). The membranes were cross-linked with UV and then blocked in 5% (w/v) skim milk in Tris-buffered saline (TBS) containing 0.1% Tween 20 (TBST) for 1 h at room temperature. Membranes were then incubated with antibodies against 5-hydroxymethylcytosine (5-hmC, 1:8000, Active Motif) overnight at 4 °C. After three washes with TBST, membranes were incubated with 1:4000 dilution of horseradish peroxidase-conjugated anti-rabbit IgG secondary antibody.^43^ Signal of dots were quantified by Image J software (NIH, Bethesda, MD, USA).

### Glucosylation of genomic 5-hmC followed by methylation-sensitive qPCR

Genomic DNA was treated with T4 Phage*β*-glucosyltransferase (T4-BGT, New England Biolabs, Ipswich, MA, USA) according to the manufacturer’s instruction. Glucosylated genomic DNA (100 ng) was digested with 10 U of *Hpa*II, *Msp*I or no enzyme (mock digestion) at 37 °C overnight, followed by inactivation for 20 min at 80 °C. The *Hpa*II- or *Msp*I-resistant fraction was quantified by qPCR using primers designed around at least one *Hpa*II/*Msp*I site, and normalizing to the mock digestion control. Resistance to *Msp*I directly translates into percentage of 5-hmC, whereas 5-mC levels were obtained by subtracting the 5-hmC contribution from the total *Hpa*II resistance.^44^ Primers used for SOCS1/153 are 5′-AGGGTCCAGAAGAGAGGGAA-3′, 5′-CCAGTCTTTTAAACCGGCTC-3′.

### Other materials and methods

Details on functional assays such as cell proliferation, apoptosis, migration and matrigel invasion assays were described in Supplementary Materials and Methods.

### Statistical analysis

Statistical analyses were performed using SPSS 16.0 for Windows (IBM, Armonk, NY, USA). Quantitative data were compared between groups using Student’s *t*-test. Categorical data were analyzed by the *χ*^2^ test or Fisher’s exact test. OS and RFS rates were calculated according to the Kaplan–Meier method, and differences were analyzed using the log-rank test. Univariate and multivariate analyses were performed using the Cox proportional hazards regression model. *P*<0.05 was considered statistically significant.

## Figures and Tables

**Table 1:** Univariate and multivariate analyses of prognostic factors in HCC (cohort 2, *n*=323)

**Variable**	**TTR**		**OS**	
	**HR (95% CI)**	***P***	**HR (95% CI)**	***P***
*Univariate analysis*				
Age, year (⩽50 *versus* >50)	0.977 (0.726–1.315)	0.879	1.207 (0.889–1.640)	0.228
Sex (female *versus* male)	1.863 (1.143–3.036)	0.013	1.757 (1.049–2.942)	0.032
HBsAg (negative *versus* positive)	0.978 (0.664–1.442)	0.912	1.002 (0.670–1.498)	0.993
AFP, ng/ml (⩽20 *versus* >20)	1.155 (0.835–1.597)	0.385	1.548 (1.083–2.211)	0.016
GGT, U/l (⩽54 *versus* >54)	1.329 (0.978–1.805)	0.069	1.737 (1.253–2.409)	0.001
Liver cirrhosis (no *versus* yes)	1.160 (0.712–1.889)	0.552	1.362 (0.801–2.315)	0.254
Tumor size, cm (⩽5 *versus* >5)	1.817 (1.346–2.452)	0.000	2.482 (1.806–3.412)	0.000
Tumor number (single *versus* multiple)	1.362 (0.907–2.044)	0.136	1.517 (1.025–2.243)	0.037
Microvascular invasion (no *versus* yes)	1.915 (1.420–2.583)	0.000	2.479 (1.815–3.388)	0.000
Tumor encapsulation (complete *versus* none)	1.228 (0.860–1.754)	0.259	1.638 (1.160–2.314)	0.005
Tumor differentiation^a^ (I+II *versus* III+IV)	1.679 (1.246–2.263)	0.001	1.715 (1.260–2.336)	0.001
TNM stage (I *versus* II–III)	1.226 (0.912–1.650)	0.178	1.563 (1.149–2.125)	0.004
5-hmC (low *versus* high)	0.709 (0.520–0.967)	0.030	0.707 (0.512–0.974)	0.034
				
*Multivariate analysis*				
Sex (female *versus* male)	1.777 (1.089–2.901)	0.021	NA	NA
AFP, ng/ml (⩽20 *versus* >20)	NA	NA	NA	NA
GGT, U/l (⩽54 *versus* >54)	NA	NA	1.458 (1.037–2.049)	0.030
Tumor size, cm (⩽5 *versus* >5)	1.722 (1.263–2.350)	0.001	2.123 (1.512–2.979)	0.000
Tumor number (single *versus* multiple)	NA	NA	NA	NA
Microvascular invasion (no *versus* yes)	1.563 (1.143–2.137)	0.005	2.137 (1.543–2.959)	0.001
Tumor encapsulation (complete *versus* none)	1.647 (1.211–2.239)	0.001	1.663 (1.207–2.291)	0.002
Tumor differentiation^a^ (I+II *versus* III+IV)	NA	NA	1.737 (1.225–2.463)	0.002
TNM stage (I *versus* II–III)^a^	NA	NA	NA	NA
5-hmC (low *versus* high)	0.634 (0.463– 0.867)	0.004	0.654 (0.472–0.906)	0.011

Abbreviations: 5-hmC, 5-hydroxymethylcytosine; AFP, alpha-fetoprotein; CI, confidential interval; GGT, gamma glutamyl transferase; HBsAg, hepatitis B surface antigen; HR, hazard ratio; NA, not adopted; TNM, tumor-node-metastasis

a Edmondson grade

Analyses were conducted using univariate analysis or multivariate Cox proportional hazards regression
