# Correction: MicroRNA-29a induces loss of 5-hydroxymethylcytosine and promotes metastasis of hepatocellular carcinoma through a TET–SOCS1–MMP9 signaling axis

**DOI:** 10.1038/s41419-024-07144-0

**Published:** 2024-11-14

**Authors:** Qing Chen, Dan Yin, Yong Zhang, Lei Yu, Xue-Dong Li, Zheng-Jun Zhou, Shao-Lai Zhou, Dong-Mei Gao, Jie Hu, Cheng Jin, Zheng Wang, Ying-Hong Shi, Ya Cao, Jia Fan, Zhi Dai, Jian Zhou

**Affiliations:** 1 Key Laboratory of Carcinogenesis and Cancer Invasion, Liver Cancer Institute, Zhongshan Hospital, Fudan University, Ministry of Education, Shanghai, 200032 China; 2 Institute of Biomedical Sciences, Fudan University, Shanghai, 200032 China; 3 Key Laboratory of Carcinogenesis and Cancer Invasion, Cancer Research Institute, Central South University, Ministry of Education, Changsha, 410078 China; 4 State Key Laboratory of Genetic Engineering, Fudan University, Shanghai, 200032 China

Correction to: *Cell Death and Disease* 10.1038/cddis.2017.142, published online 29 June 2017

The original version of this article unfortunately contained two incorrect images in the Figure 1e and Figure 4f. The online version of these Figures has been updated with the correct images. The correction does not affect the conclusion of the article. The authors apologize for the errors.


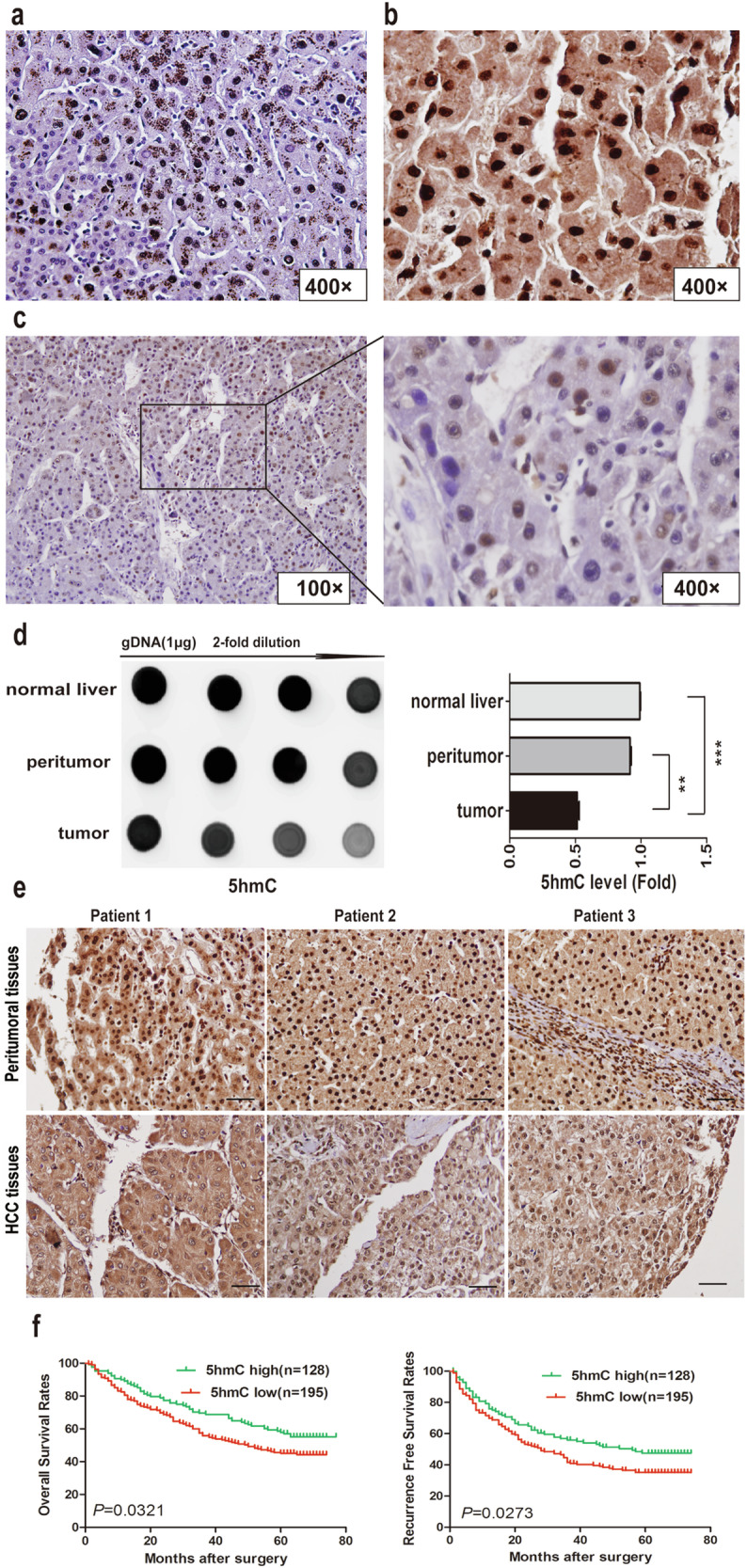


Figure 1
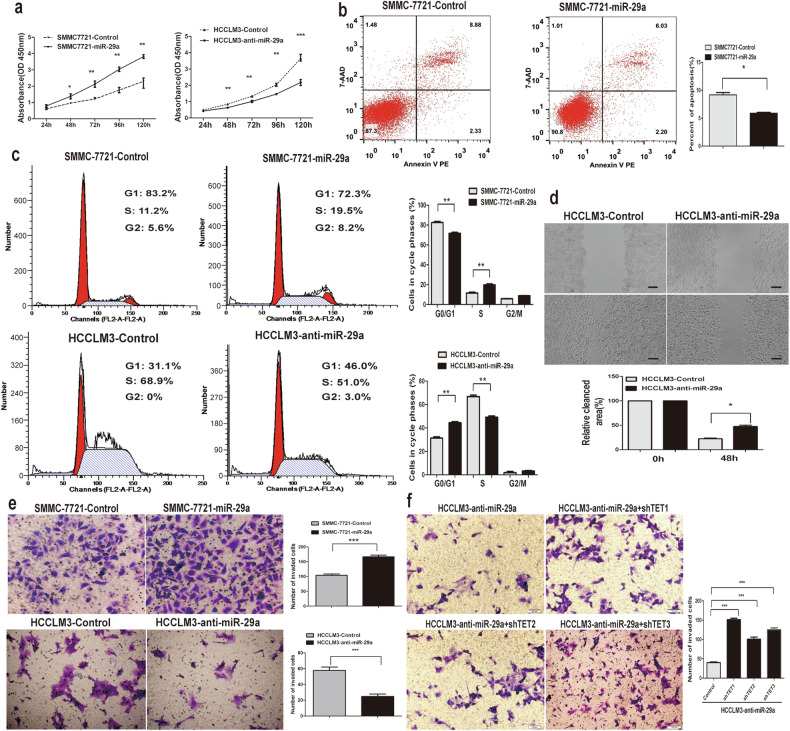


Figure 4

