# Supplementary material for: MicroRNA-29a induces loss of 5-hydroxymethylcytosine and promotes metastasis of hepatocellular carcinoma through a TET–SOCS1–MMP9 signaling axis
Source: Cell Death Dis. 2017 Jun 29;8(6):e2906–. doi: 10.1038/cddis.2017.142 (PMC5520877; doi:10.1038/cddis.2017.142)
Supplement: Supplementary Figure Legends [file cddis2017142x1.docx]

**Supplementary Figure legends**

**Supplementary Figure S1.** **Downregulation of TET family members is related to the loss of 5-hmC.** (A) Fluorescence microscopic analysis for TET family member expression and 5-hmC levels in tumor and corresponding nontumor tissues. HCC tissues (denoted as T) displayed weaker immunostaining for TET family members especially in the cytoplasm, and for 5-hmC in the nucleus compared with corresponding nontumor tissues (denoted as P), which displayed strong fluorescence. (B) Scoring system of 5-hmC levels by IHC staining, Related to Figure 1. Images represent the 5-hmC level scoring system for IHC staining based on 5-hmC-positive cell counts: 0=Negative (<1% tumor cells immunoreactive), 1+=Low positive (<10% tumor cells immunoreactive), 2+=Positive (10–24% tumor cells immunoreactive), 3+=Positive (25–74% tumor cells immunoreactive), and 4+=Positive (>74% tumor cells immunoreactive). (C)IDH1/2 expression levels in HCC and corresponding nontumor tissues. IDH1/2 expression was analyzed using qRT-PCR with HCC patient samples RNAs. Comparing differences in the expression levels of IDH1 and IDH2 between tumor and corresponding nontumor tissues. Data are shown as mean ± SD (n=3). Statistical significance was assessed by Student’s *t*-test. (D) IHC staining analysis for TET family member expression in HCC and corresponding nontumor tissues. HCC tissues displayed weaker immunostaining for all TET Family members especially in the cytoplasm compared with corresponding nontumor tissues. *Scale bars*=100µm.

**Supplementary Figure S2. miR-29a targets ten eleven translocation enzymes.** (A) Putative miR-29a target sites in the TET1/2/3 3’-UTR. The seed sequences of predicted miR-29a binding sites in the indicated human genes are shown using bolded text. Point mutation made to abolish the potential pairing in the seed regions are shown using text. (B) Location of miR-29a target sites in the TET1/2/3 3’-UTR.TargetScan predicted potential miR-29a target sites in the TET1/2/3 3’-UTR are shown as black rectangles. Sites within cooperative distance of each other (within 45 nt, but no closer than 8 nt) are indicated (square brackets). Dre, zebrafish; Hsa, human; Mmu, mouse. (C) qRT-PCR confirmed different miR-29a expression in HCC cell lines and the nontransformed hepatic cell line L-02. Data are shown as mean ± SD (n=3). Statistical significance was assessed by Student’s *t*-test.

**Supplementary Figure S3. Modifications of miR-29a levels in HCC cells with different baseline miR-29a levels.** (A, B) Following transfection with anti-miR-29a vector, the miR-29a level was significantly downregulated, while both the mRNA and protein levels of TET1, 2, and 3 in MHCC97H and HCCLM3 cells were significantly increased compared with controls. (C) Genomic DNA was purified from HCCLM3 cells expressing anti-miR-29a vector or control miRNA. Global 5-hmC levels were measured using anti-5hmC dot blot assay. (D) Modulation of miR-29a was confirmed on a functional level by checking the protein expression of PTEN, an experimentally validated miR-29a target. PTEN was remarkably reduced in HepG2 and SMMC-7721 cells when miR-29a was upregulated and significantly increased when miR-29a was downregulated in HCCLM3 and MHCC97H cells. Data are shown as mean ±SD (n=3). Statistical significance was assessed by Student’s *t*-test.

**Supplementary Figure S4.The effects of miR-29a on in vitro migration, and apoptosis of HCC cells.** (A) Knockdown of miR-29a in HCCLM3 cells significantly increased the apoptosis rate compared with control cells. (B) Upregulation of miR-29a in SMMC-7721 significantly increased wound healing compared with control cells. Data are shown as mean ±SD (n=3). Statistical significance was assessed by Student’s *t*-test; **P*<0.05, ***P*<0.01; *Scale bar*=100 μm.

**Supplementary Figure S5**. **Tumor suppressor genes expression was altered in HCC cells transfected with miR-29a and anti-miR-29a vector.** (A) qRT-PCR was used to detect the expression of 8 different tumor suppressor genes including APC, RASSF1A, SOCS1, HIC1, GSTP1, CDKN2A, RUNX3, and PRDM2 in SMMC-7721-miR-29a and HCCLM3-anti-miR-29a cells. The results showed many tumor suppressor genes RNAs were altered. SOCS1 ranked first among them. (B) GlucMS-qPCR analysis of CpG islands within the SOCS1 promoter regions specifically enriched for 5-hmC in HepG2 cells infected with miR-29a expressing vector. (C) SOCS1 expression was restored when treated with DNA-demethylating agent 5’-aza-2’-deoxycytidine in HepG2 cells transfected with miR-29a expression vector. Data are shown as mean ± SD (n=3). Statistical significance was assessed by Student’s *t*-test.

**Supplementary Figure S6. miR-29a targets DNMT3A, DNMT3B and has no effect on 5hmC levels.** (A) western blot analysis showed a significant DNMT1, DNMT3A, DNMT3B, and SOCS1 protein expression decreased in highly invasive HCCLM3 and MHCC97H cells compared with SMMC-7721 and HepG2 cells. (B) Following transfection with miR-29a vector, the protein levels of DNMT3A and DNMT3B in SMMC-7721 and HepG2 cells were significantly decreased compared with controls. (C)shDNMT1, shDNMT3A, and shDNMT3B in HCCLM3-anti-miR-29a cells were determined by western blot analysis (left). Genomic DNA purified from HCCLM3-anti-miR-29a cells with shDNMT1, DNMT3A, DNMT3B and negative control. Global 5-hmC levels were measured using an anti-5hmC dot blot assay (right). (D) Invasive behavior was tested using Transwell Matrigel invasion assays after shDNMT1, shDNMT3A, and shDNMT3B in HCCLM3-anti-miR-29a cells (left). Quantitation of tumor cell invasion is shown (right). (E) shDNMT1, shDNMT3A, and shDNMT3B in HCCLM3-anti-miR-29a cells, the apoptosis rate were determined by flow cytometry. (F) DNMT3A expression is weakly negative correlation to miR-29a expression in human HCC tissues. The correlation between miR-29a and DNMT3A expressions was analyzed by using a real-time qPCR with RNAs from HCC patient samples. Data are shown as mean ± SD (n=3). Statistical significance was assessed by Student’s *t*-test.
