# Supplementary figures and images for: MicroRNA-29a induces loss of 5-hydroxymethylcytosine and promotes metastasis of hepatocellular carcinoma through a TET–SOCS1–MMP9 signaling axis
Source: Cell Death Dis. 2017 Jun 29;8(6):e2906–. doi: 10.1038/cddis.2017.142 (PMC5520877; doi:10.1038/cddis.2017.142)

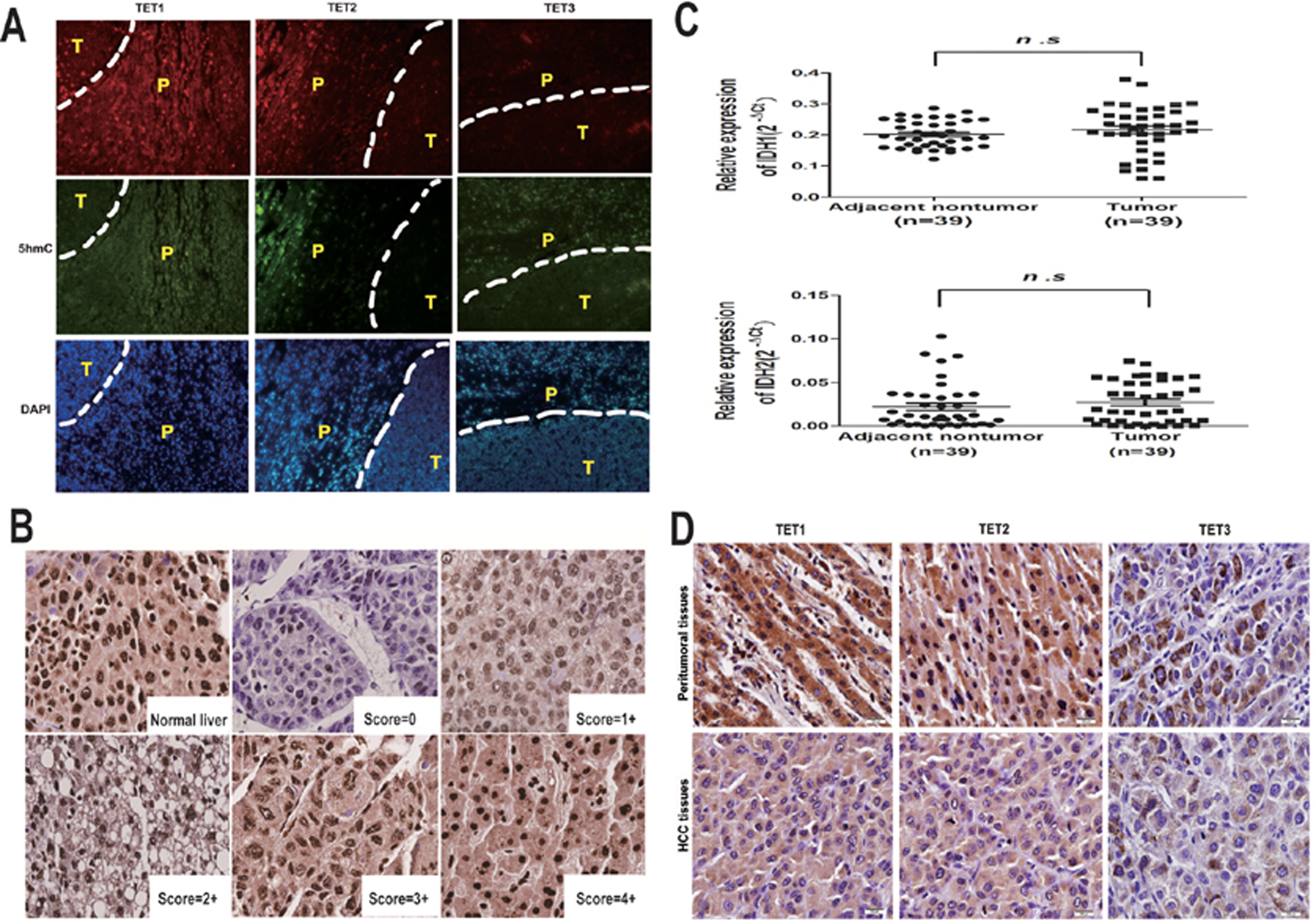

Supplement: Supplementary Figure S1 [file cddis2017142x2.tif]
