# Supplementary material for: MicroRNA-29a induces loss of 5-hydroxymethylcytosine and promotes metastasis of hepatocellular carcinoma through a TET–SOCS1–MMP9 signaling axis
Source: Cell Death Dis. 2017 Jun 29;8(6):e2906–. doi: 10.1038/cddis.2017.142 (PMC5520877; doi:10.1038/cddis.2017.142)
Supplement: Supplementary Tables 1-4 [file cddis2017142x8.docx]

**Supplementary Tables**

**Supplementary Table S1. Correlation between the Factors and Clinicopathologic Characteristics in HCC (Cohort 1, n =323)**

| **Clinicopathological Indexes** | | **5hmC** | | ***P*** |
| --- | --- | --- | --- | --- |
|  |  | **low** | **high** |  |
| Age(year) | ≤50  >50 | 103  92 | 63  65 | 0.526 |
| Sex | Female  Male | 32  163 | 14  114 | 0.169 |
| HBsAg | Negative  Positive | 24  171 | 21  107 | 0.298 |
| HCV | Negative  Positive | 193  2 | 123  5 | 0.118* |
| AFP (ng/ml) | ≤20  >20 | 52  143 | 44  84 | 0.138 |
| GGT (U/L) | ≤54  >54 | 76  119 | 55  73 | 0.475 |
| Liver cirrhosis | No  yes | 23  172 | 14  114 | 0.813 |
| Tumor size(cm) | ≤5  >5 | 100  95 | 64  64 | 0.822 |
| Tumor number | Single  Multiple | 164  31 | 112  16 | 0.397 |
| Microvascular invasion | absence | 102  93 | 73  55 | 0.405 |
| Tumor encapsulation | present complete none | 99  96 | 65  63 | 0.998 |
| Tumor differentiation | I+II  III+IV | 152  43 | 104  24 | 0.474 |
| TNM stage^a^ | I  II+III | 88  107 | 76  52 | 0.012 |

AFP, alpha-fetoprotein; GGT, gamma glutamyl transferase; HBsAg, hepatitis B surface antigen; 5hmC, 5-hydroxymethylcytosine; TNM, tumor-node-metastasis.

*Fisher’s exact tests; chi-square tests for all other analyses.

a Edmondson grade.

**Supplementary Table S2. The primer sequences of human RNAs for real-time PCR.**

| **Primer name and sequences** | |
| --- | --- |
| TET1 | Forward: 5’-TGAGGAATCAGAGCAGAGAACA-3’ |
|  | Reverse: 5’-AGTTTGGGTCTTGGAGGTCTTT-3’ |
| TET2 | Forward: 5’-GCAAAACCTGTCCACTCTTATG-3’ |
|  | Reverse: 5’-CACTCTGGTGCTCTGTGTTCAT-3’ |
| TET3 | Forward: 5’-CGTCTTAGTCATCTCCCCAGTG-3’ |
|  | Reverse: 5’-GAGCAGAAAAGGAACCAAATGT-3’ |
| GAPDH | Forward: 5’-AAGGTGAAGGTCGGAGTCAAC -3’ |
|  | Reverse: 5’-GGGGTCATTGATGGCAACAATA-3’ |
| IDH1 | Forward: 5’-CCACCAACGACCAAGTCAC-3’ |
|  | Reverse: 5’-GAACTCCTCAACCCTCTTCTCA-3’ |
| IDH2 | Forward: 5’-AAAGATGGCAGTGGTGTCAAG-3’ |
|  | Reverse: 5’-CGCAAAACCTGAGATGGACTC-3’ |
| MMP9 | Forward: 5’-GAACTTTGACAGCGACAAGAAGT-3’ |
|  | Reverse: 5’-AGTGAAGCGGTACATAGGGTACA-3’ |
| SOCS1 | Forward: 5’-TGGTTGTAGCAGCTTGTGTCTGG-3’ |
|  | Reverse: 5’-CCTGGTTTGTGCAAAGATACTGGG-3’ |
| APC | Forward: 5’-GTCAATACCCAGCCGACCTA-3’ |
|  | Reverse: 5’-TGCCCATCTTTCATTCTGTG-3’ |
| RASSF1A | Forward: 5’-CAACTCCTCACACACCCTGA-3’ |
|  | Reverse: 5’-GAGACCCTGGCTTTGATTAGC-3’ |
| HIC1 | Forward: 5’-GCCGCTCCAGATAAGAGTGT-3’ |
|  | Reverse: 5’-CTCCATCGTGTCCAGCATC-3’ |
| GSTP1 | Forward: 5’-CCGTGGTCTATTTCCCAGTTC-3’ |
|  | Reverse: 5’-AGGTGACGCAGGATGGTATT-3’ |
| CDKN2A | Forward: 5’-AAACACCGCTTCTGCCTTT-3’ |
|  | Reverse: 5’-CCCTGAGCTTCCCTAGTTCAC-3’ |
| RUNX3 | Forward: 5’-TGCACTTTACCAGCTCAACG-3’ |
|  | Reverse: 5’-CCCTCCTGTTCTCTCCACAA-3’ |
| PRDM2 | Forward: 5’-GCACCGTCTACCCTTCTCTG-3’ |
|  | Reverse: 5’-GAAGAACACGTCCAAGCACA-3’ |
| PTEN | Forward: 5’-TGCAGAGTTGCACAATATCC-3’ |
|  | Reverse: 5’-CTGAGGATTGCAAGTTCCGCC-3’ |
| DNMT3A | Forward: 5’-ATAAAGCAGGGCAAAGACCA-3’ |
|  | Reverse: 5’-GGACTGGGAAACCAAATACC-3’ |

**Supplementary Table S3. Suppressor of cytokine signaling 1 (SOCS1) promoter primers used in the chromatin immunoprecipitation (ChIP) assay.**

| **Primer Number** | **Sequence** |
| --- | --- |
| **1** | Forward: 5’- ACTCCAGCCTGAGCAACA-3’ |
|  | Reverse: 5’- GCAGTGAGCCACGATTGT-3’ |
| **2** | Forward: 5’- AGCCTTGACCTCCTGGGCT-3’ |
|  | Reverse: 5’- ATTAACCTCTCTGCCATG-3’ |
| **3** | Forward: 5’- TACATGCTCAAGGTCACA-3’ |
|  | Reverse: 5’- CAGGGAGTGGCCCAGAAT-3’ |
| **4** | Forward: 5’- TGTTCAGGAGGTTAATAAG-3’ |
|  | Reverse: 5’- CGTGTTTGTGATTTAGTG-3’ |
| **5** | Forward: 5’- TGTGCAAATTACAGCCCCGACCA-3’ |
|  | Reverse: 5’- TGCAATGGCTTGCAGATTGGGAG-3’ |
| **6** | Forward: 5’- AATCCCAGCCCCTCCCCA-3’ |
|  | Reverse: 5’- CCCACCCGTAGACCCCCT-3’ |
| **7** | Forward: 5’- GTCAGGCTCAGGGTTGGGGA-3’ |
|  | Reverse: 5’- GGGGCCCTCTGCCCGCCT-3’ |
| **8** | Forward: 5’- GCGGGAGGGTCCAGAAGAGA-3’ |
|  | Reverse: 5’- CTGGCGGCGGGGCGCGGG-3’ |
| **9** | Forward: 5’- GTGAGCCGGGCCCTGGGC-3’ |
|  | Reverse: 5’- GGGGGCGTGGAGAGCAGC-3’ |
| **10** | Forward: 5’- CTCTCGGTGCTGCCCGGA-3’ |
|  | Reverse: 5’- CCTACAGAAGGGGCCAGC3’ |

**Supplementary Table S4. The siRNA sequences used in this study.**

| **siRNA name and sequences** | | |
| --- | --- | --- |
| TET1 | sense | 5’-CCAGUCUUAAUAAGGUUAUdTdT-3’ |
|  | antisense | 5’-AUAACCUUAUUAAGACUGGdTdT-3’ |
| TET2 | sense | 5’-GGGUAAGCCAAGAAAGAAAdTdT-3’ |
|  | antisense | 5’-UUUCUUUCUUGGCUUACCCdTdT-3’ |
| TET3 | sense | 5’-GAGGCUGAAUUUGGAGAUAdTdT-3’ |
|  | antisense | 5’-UAUCUCCAAAUUCAGCCUCdTdT-3’ |
| dnmt1 | sense | 5’-GGAAGUGAAUGGACGUCUATT-3’ |
|  | antisense | 5’-UAGACGUCCAUUCACUUCCCG-3’ |
| dnmt3a | sense | 5’-agccaucuacgagguccugTT-3’ |
|  | antisense | 5’-caggaccucguagauggcuTT-3’ |
| dnmt3b | sense | 5’-GCUCUUACCUUACCAUCGATT-3’ |
|  | antisense | 5’-UCGAUGGUAAGGUAAGAGCTG-3’ |
| negative control | sense | 5’-UUC UCC GAA CGU GUC ACG UTT-3’ |
|  | antisense | 5’-ACG UGA CAC GUU CGG AGA ATT-3’ |
