# Supplementary material for: MicroRNA-29a induces loss of 5-hydroxymethylcytosine and promotes metastasis of hepatocellular carcinoma through a TET–SOCS1–MMP9 signaling axis
Source: Cell Death Dis. 2017 Jun 29;8(6):e2906–. doi: 10.1038/cddis.2017.142 (PMC5520877; doi:10.1038/cddis.2017.142)
Supplement: Supplementary Materials and Methods [file cddis2017142x9.docx]

**Supplementary Materials and Methods**

***Patients and Follow-up.***

Two independent cohorts of 431 HCC patients were enrolled in this study. The 108 tumor tissues and paired adjacent nontumor cirrhotic liver tissues used in RT-qPCR analysis were consecutively collected from patients undergoing curative resection from January to December 2007 (cohort 1, snap-frozen tissues). Paraffin-embedded tissues from cohort 2 were randomly obtained from HCC patients undergoing curative resection from 2003 to 2004 at the Liver Cancer Institute, Zhongshan Hospital, Fudan University (cohort 2, *n*=323). Patients in cohort 1 were monitored after surgery, until March 15, 2013, while Patients in cohort 2 were monitored until March 15, 2009.The histopathological diagnose were based on the World Health Organization criteria. The histological grade of tumor differentiation was determined according to the classification proposed by Edmondson and Steiner.^1^ Liver function was assessed by the Child-Pugh scoring system. Tumor stage was determined according to the 2010 International Union against Cancer tumor-node-metastasis classification system. Ethical approval for the use of human subjects was obtained from the Research Ethics Committee of Zhongshan Hospital, and informed consent was obtained from each patient. Postsurgical patient surveillance was performed as previously described.^2, 3^ Overall survival (OS) was defined as the interval between surgery and death or between surgery and the last observation point. For surviving patients, the data were censored at the last follow-up. Time to recurrence (TTR) ^4^ was defined as the interval between the date of surgery and the date of any diagnosed relapse (intrahepatic recurrence and extrahepatic metastasis).

***Chromatin Immunoprecipitation (ChIP)-PCR Analysis.***

ChIP was performed in native conditions. Briefly, cells at a concentration

of 2 million cells/mL were treated with 1% formaldehyde in medium for 10 mins

 at room temperature.  After being washed twice with ice-cold PBS containing protease inhibitors, cells were pelleted by centrifugation and resuspended in SDS lysis buffer. After incubation for 15 min at 4°C, the lysates were sonicated 12 times (30 sec each) using a Branson Sonifier 450 (Branson Ultrasonics, Danbury, CT, USA). After centrifugation, the supernatant was diluted in ChIP dilution buffer and incubated overnight at 4°C with TET1 antibody (61741; Active motif) and protein G beads. Samples were washed two times in lysis buffer, four times in 1M lysis buffer (50 mM Tris, pH 7.4, 1 M NaCl, 1 mM EDTA, 0.1% SDS, 1% NP-40, and 0.5% sodium

deoxycholate), the beads were then resuspended in lysis buffer and treated with

proteinase K at 45 ºC for 45 min. Co-precipitated DNAs were purified using QIAquick DNA purification spin column (Qiagen, Germantown, MD, USA) and eluted in 50 µL nuclease-free water. The immunoprecipitated DNA was quantified using PCR, and all values were normalized to the input. ChIP-PCR analysis primers are shown in Supplementary Table S2.

***Vectors and Cell Transfection.***

The following miRNA vectors were purchased from Shanghai GeneChem Co.: miR-29a expression vector (Ubi-EGFP-MCS-IRES-Puromycin), the control vector for miR-29a (Ubi-EGFP-MCS-IRES-Puromycin), miR-29a inhibitor (hU6-MCS-Ubiquitin-EGFP-IRES-puromycin), and the negative control for the miR-29a inhibitor (hU6-MCS-Ubiquitin-EGFP-IRES-puromycin). Ubi-EGFP-MCS-IRES-Puromycin -miR-29a was transfected into HCC cells with lower metastatic potential (HepG2 and SMMC-7721) and hU6-MCS-Ubiquitin-EGFP-IRES-puromycin-mir-29a was transfected into highly metastatic HCC cells (MHCC97H and HCCLM3). Ubi-EGFP-MCS-IRES-Puromycin and hU6-MCS-Ubiquitin-EGFP-IRES-puromycin lentiviral vectors were used as controls. Stably transfected clones were validated by RT-qPCR and immunoblotting for TET1, 2, and 3. Modulation of miR-29a function was verified by examining the protein expression of PTEN, a well-established miR-29a target.^5^ As shown in Supplementary Fig. S3D. The target shRNA sequences are listed in the Supplementary Table S4.

The wild-type 3’-UTR segment of human TET1, 2, and 3 mRNA, which contained a putative binding site for miR-29a, was amplified from normal human genomic DNA and inserted into the NotI/XhoI sites downstream of the stop codon of *Renilla* luciferase in the psi-check2 vector (Promega, Madison, WI). The psi-check2 vector is a dual-luciferase reporter plasmid that contains the firefly luciferase and the *Renilla* luciferase reporter genes. The mutant 3'-UTR of TET1, 2, and 3 contained a mutated sequence in the complementary site for the seed region of miR-29a (Supplementary Fig. S2*A*). All constructs were verified by direct sequencing. Cells were transfected with the vectors mentioned above using Lipofectamine 2000 according to the manufacturer’s instructions (Invitrogen, Carlsbad, CA).

***Cell Proliferation, Cell Migration, and Matrigel Invasion Assays.***

Cells (2000 cells/well) were dispensed in 100 μL aliquots into a 96-well plate. At the indicated time points, 10 μL CCK-8 solution (Dojindo) was added to the cells, and plates were incubated for an additional 2 hours. The absorbance at 450 nm was measured to determine the number of viable cells in each well. All experiments were performed three times.

Cell migration was evaluated using the scratch wound assay. Cells were cultured for 2 days to yield a tight cell monolayer and then serum starved for 16 hours. After serum starvation, the cell monolayer was wounded with a 10 μL plastic pipette tip. The remaining cells were washed twice with culture medium to remove cell debris and incubated at 37°C with normal serum-containing culture medium. At the indicated times, migrating cells at the wound front were photographed using an inverted microscope (Leica). The percentage of cleared area at each time point compared with time zero was measured using Image-Pro Plus v6.2 software.

Cell invasion assays were performed using 24-well Transwells plates (8-μm pore size; Minipore) precoated with Matrigel (BD Biosciences, Franklin Lakes, NJ). In total, 1×10^5^ cells were suspended in 100 μL Dulbecco’s modified Eagle medium with 1% fetal bovine serum and were added to the upper chamber, and 600 μL Dulbecco’s modified Eagle medium with 10% fetal bovine serum was placed in the lower chamber. After 48 hours of incubation, the Matrigel and the cells remaining in the upper chamber were removed with cotton swabs. Cells on the lower surface of the membrane were fixed in 4% paraformaldehyde and stained with Crystal Violet. Cells in five microscopic fields (at 200× magnification) were counted and photographed. All experiments were performed in triplicate.

***In Vivo Assays for Tumor Growth and Metastasis.***

SMMC-7721-miR-29a, SMMC-7721-control, HCCLM3-anti-miR-29a, and HCCLM3- control cells (5×10^6^) were suspended in 100 μL serum-free Dulbecco’s modified Eagle medium and Matrigel (BD Biosciences) at a (1:1 ratio) and then injected subcutaneously into the upper left flank region of nude mice (3 in each groups). When the subcutaneous tumor reached approximately 1 cm in length (approximately 4 weeks after injection), it was removed, minced into small pieces of equal volume (2×2×2 mm^3^), and transplanted into the livers of 32 different nude mice (8 in each groups). All mice were monitored once every 3 days and sacrificed 5 weeks later. The volume of tumors was calculated in mm^3^ as follows: V= ab^2^⁄2 (with a and b representing the largest and smallest tumor diameters measured at necropsy, respectively).^6^

Lungs were removed and embedded in paraffin and the total number of lung metastases was examined under the microscope as described previously.^7^ The metastases were classified into four grades on the basis of the number of tumor cells present at the maximal section for each metastatic lesion: grade I, ≤20 tumor cells; grade II, 20-50 tumor cells; grade III, 50-100 tumor cells; and grade IV, >100 tumor cells. Tumor tissue sections were prepared, and immunoreactivity was analyzed as above using antibodies to -TET1 (1:150, clone ab191698, Abcam), TET2 (1:100, clone ab94580, Abcam), TET3 (1:200, clone ab139805, Abcam), 5-hmC (1:500, Catalog No.39769, Active Motif), p-ERK1/2 (Thr202/Tyr204) and p-STAT3 (Tyr705), (1:150, Cell Signaling Technology), SOCS1 (1:50, clone ab83493, Abcam), PTEN(1:100, clone ab31392, Abcam), Ki-67 (1:100, clone ab16667,Abcam), and MMP9 (1:400, clone ab38898, Abcam).

***Evaluation of IHC Variables.***

IHC staining was assessed by three independent investigators who were blinded to patient characteristics. Discrepancies were resolved by consensus. Under high-power magnification (400 or 200×), photographs of three representative fields were captured by the Leica QWin Plus v3 software using identical settings for each photograph. The 5-hmC density was determined with the Image-Pro Plus v6.2 software (Media Cybernetics, Inc., Bethesda, MD). Integrated optical densities of all positive 5-hmC staining in each photograph were measured, and the ratio of this value to the total area of each photograph was calculated and deemed the 5-hmC density. We utilized a scoring system for 5-hmC levels by IHC staining that was related to representative histology for cell counts of 5-hmC positive immunoreactivity as described previously.^8^ The 5-hmC immunostaining intensities were semiquantitatively scored as follows: 0=Negative (<1% tumor cells immunoreactive), 1+=Low positive (<10% tumor cells immunoreactive), 2+=Positive (10–24% tumor cells immunoreactive), 3+=Positive (25–74% tumor cells immunoreactive), and 4+=Positive (>74% tumor cells immunoreactive), as shown in Supplementary Fig. S1*B*. When 5-hmC was analyzed using IHC, the 5-hmC density staining score ≥2 was used as a cut-off for subsequent analyses. HCC patients with a 5-hmC density higher (staining score≥2) or lower (staining score<2) than the median 5-hmC density were defined as 5-hmC high or 5-hmC low, respectively.

**REFERENCES**

1. Wittekind C. [Pitfalls in the classification of liver tumors]. Pathologe 2006;27:289-293.
2. Sun HC, Zhang W, Qin LX, Zhang BH, Ye QH, Wang L, et al. Positive serum hepatitis B e antigen is associated with higher risk of early recurrence and poorer survival in patients after curative resection of hepatitis B-related hepatocellular carcinoma. J Hepatol 2007;47:684-690.
3. Yang XR, Xu Y, Shi GM, Fan J, Zhou J, Ji Y, et al. Cytokeratin 10 and cytokeratin 19: predictive markers for poor prognosis in hepatocellular carcinoma patients after curative resection. Clin Cancer Res 2008;14:3850-3859.
4. Llovet JM, Di Bisceglie AM, Bruix J, Kramer BS, Lencioni R, Zhu AX, et al. Design and endpoints of clinical trials in hepatocellular carcinoma. J Natl Cancer Inst 2008;100:698-711.
5. Kong G, Zhang J, Zhang S, Shan C, Ye L, Zhang X. Upregulated microRNA-29a by hepatitis B virus X protein enhances hepatoma cell migration by targeting PTEN in cell culture model. PLoS One 2011;6:e19518.
6. Wang L, Tang ZY, Qin LX, Wu XF, Sun HC, Xue Q, et al. High-dose and long-term therapy with interferon-alfa inhibits tumor growth and recurrence in nude mice bearing human hepatocellular carcinoma xenografts with high metastatic potential. Hepatology 2000;32:43-48.
7. Tian J, Tang ZY, Ye SL, Liu YK, Lin ZY, Chen J, et al. New human hepatocellular carcinoma (HCC) cell line with highly metastatic potential (MHCC97) and its expressions of the factors associated with metastasis. Br J Cancer 1999;81:814-821.
8. Lian CG, Xu Y, Ceol C, Wu F, Larson A, Dresser K, et al. Loss of 5-hydroxymethylcytosine is an epigenetic hallmark of melanoma. Cell 2012;150:1135-1146.
